# Association between nutrient intake related to the one-carbon metabolism and colorectal cancer risk: a case–control study in the Basque Country

**DOI:** 10.1007/s00394-023-03229-2

**Published:** 2023-08-06

**Authors:** Silvia Martín-Fernández-de-Labastida, Iker Alegria-Lertxundi, Marian M. de Pancorbo, Marta Arroyo-Izaga

**Affiliations:** 1 Department of Pharmacy and Food Sciences, Faculty of Pharmacy, University of the Basque Country UPV/EHU, 01006 Vitoria-Gasteiz, Araba/Álava, Spain; 2 Department of Z. and Cellular Biology A., Faculty of Pharmacy, University of the Basque Country UPV/EHU, 01006 Vitoria-Gasteiz, Araba/Álava, Spain; 3 BIOMICs Research Group, Microfluidics & BIOMICs Cluster, Lascaray Research Center, University of the Basque Country UPV/EHU, 01006 Vitoria-Gasteiz, Araba/Álava, Spain; 4 Bioaraba, BA04.03, Vitoria-Gasteiz, Araba/Álava, Spain

**Keywords:** Colorectal cancer, Nutrients, One-carbon metabolism, Diet, Protective factors, Case–control study

## Abstract

**Purpose:**

Epidemiologic evidence for the association between methyl-donor nutrient intake and colorectal cancer (CRC) risk remains inconclusive. We aimed to examine the relationship between intake of vitamins of the B group, methionine, total choline and betaine and CRC risk, in a population from the CRC screening programme in the Basque Country.

**Design:**

This observational study included 308 patients with CRC and 308 age- and sex-matched subjects as controls. During recruitment, dietary, anthropometric, lifestyle, socioeconomic, demographic, and health status information was collected. Conditional logistic regression was used to estimate the odds ratios (ORs) for CRC risk.

**Results:**

The adjusted ORs for CRC risk decreased with higher intakes of choline and betaine (*p* < 0.05). After further adjustment for folate, high intake of choline and betaine remained associated with a reduced CRC risk (adjusted model for choline, OR third tertile vs first tertile = 0.45, 95% CI 0.26–0.80, *p* = 0.006; for betaine, OR third tertile vs first tertile = 0.27, 95% CI 0.16–0.47, *p* < 0.001). Regarding the other nutrients, our findings indicated a non-significant decrease in CRC risk with the high level of intake.

**Conclusions:**

Our data suggest that choline and betaine intake influence CRC risk in the studied population.

**Supplementary Information:**

The online version contains supplementary material available at 10.1007/s00394-023-03229-2.

## Introduction

Colorectal cancer (CRC) is the third most frequent cancer and the second highest mortality in cancer patients worldwide [1]. Global cancer statistics in 2020 has shown there are about 1.93 million new cases and 935,000 deaths of CRC worldwide, accounting for 10.0% of the total new cases of cancer and 9.4% of the total cancer-related deaths, respectively [1].

During recent years, mortality rates for CRC have been decreasing due to early screening programmes [2, 3] and better treatment options [4]. However, the aetiology of CRC is complex and still not fully understood. Both genetic and environmental factors play an important role in the aetiology of this disease [5]. Large population studies with varying strengths of evidence have found CRC-protective factors such as certain nutrients involved in one-carbon metabolism (OCM), that is, B vitamins, methionine (Met), choline and betaine, among others [6, 7].

A folate metabolite, 5-methyl-tetrahydrofolate (THF), provides the methyl group in the reaction by methionine synthase (MTR) to convert homocysteine (Hcy) to Met, the precursor of the universal methyl donor S-adenosyl-Met, which is involved in epigenomic regulatory mechanisms. Thus folate/methyl depletion results in aberrant DNA methylation, i.e., global genomic hypomethylation and specific methylation of CpG clusters in the promoters of tumour suppressor and DNA repair genes [8]. CpG cluster or island is defined as a 200-bp region of DNA with a GC content higher than 50% and an observed CpG versus expected CpG ratio greater or equal to 0.6 [9]. CpG islands are often present around the promotors of housekeeping genes and other actively expressed genes.

These important cellular processes are related to folate metabolism linked by methylentetrahydrofolate reductase (MTHFR) which catalyses the irreversible conversion of 5,10-methylene-THF to 5-methyl-THF. The MTHFR substrate, 5,10-methylene-THF, is also a substrate for the thymidylate synthase enzyme in the methylation of deoxyuridine monophosphate to deoxythymidine monophosphate, with is the sole de novo source of thymidine and the rate-limiting step in DNA synthesis in mammalian cells [8].

In this context, the roles of B vitamins in OCM are the following: vitamin B_2_ is the precursor for a cofactor of MTHFR [10]; vitamin B_6_ is not only a cofactor for cystathionine-beta-synthase to convert Hcy to cystathionine but also is required in the conversion of serine to glycine, recycling THF (derivative of 5-methyl-THF after methyl-transfer to Hcy) to 5, 10-methylene-THF [11]; vitamin B_12_ is a cofactor of MTR in the synthesis of Met [12]. Choline is a necessary source of methyl groups for methyl group transfer and can be oxidized to betaine to participate in DNA methylation, especially in folate-deficiency populations. A deficiency of methyl-donors might lead to the disruption of DNA methylation and impaired DNA repair [13], which could promote colorectal carcinogenesis [14]. Alcohol, for its part, is also known to play an important role in folate metabolism, being a folate antagonist and an inhibitor of MTR [15].

Considering OCM nutrients need to be obtained from food, several epidemiological studies have assessed the association between some methyl-donor nutrient intake and the risk of CRC, however, the results remained inconclusive. Some of them reported an inverse association, but not all, between folate intake [16–18], vitamin B_2_, vitamin B_6,_ and vitamin B_12_ and CRC risk [19–21]. In addition, low Met intake, especially in combination with low folate and high alcohol intake, was related to an increased risk of CRC [22, 23], although Met intake was unrelated to CRC in other studies [24]. High choline intake has been related to decreased risk of CRC in a case–control study conducted in China [25], whereas a cohort study has failed to find a protective association between choline and betaine intake and CRC risk in USA [26]. These inconsistencies may be due, in part, to differences in nutrient intake between populations.

The prevalence of adequacy for folate intake in the Spanish population according to the national criteria, the Recommended Dietary Intakes, is very low (3.0% in women and 6.6% in men) [27]. Whereas, alcohol consumption is frequent among Spanish older adults [28], which may further compromise folate and, more generally, methyl status in this population. Furthermore, CRC incidence in Spanish population is comparable to that found in high-risk zones of Eastern Europe, Russia, and Japan [29], and, in particular, some Spanish autonomous regions such as the Basque Country, have an incidence of this type of cancer above the national average [30].

For all the above reasons, that is, the inconsistencies in the associations between methyl-donor nutrient intake and the risk of CRC, and the high incidence of this type of cancer in the Basque Country (a Spanish autonomous region located in the North of the country), the objective of the present study was to examine associations between intakes of nutrients related to OCM (in particular, folate, vitamins of the B group (B_2_, B_3_, B_6_, B_12_), Met, betaine and total choline) and CRC risk in a sample of cases and controls from the population-based bowel cancer screening programme (BCSP) of the Osakidetza/Basque Health Service. We hypothesised that the nutrients mentioned above were inversely associated with CRC risk. The result of this study might be beneficial for CRC prevention by improving nutritional behaviour.

## Methods

Overall, this epidemiologic study is an observational analytic case–control study designed to address possible gene–diet interaction in relation to CRC, in particular, to analyse the relationship between diet and the methylation status in CpG islands in colorectal tumours.

### Sampling and study subjects

Participants in this study were recruited from among patients attending any of the following hospitals of the Osakidetza/Basque Health Service, members at the same time of the Basque Country’s BCSP: Basurto, Galdakao, and Donostia. To be eligible for this BCSP, the patients had to be aged between 50 and 69, asymptomatic for colorectal symptoms, and registered with the Osakidetza/Basque Health Service [31]. These inclusion criteria were applied to both case and control groups; that is, controls fulfilled the same eligibility criteria defined for the cases, with the exception of the disease (outcome). The sample size was estimated to be 286 in each group to detect an odds ratio (OR) of 2.0 with 80% power at a two-sided level of significance of 5%, under an exposure prevalence of 10%, using the Epidat 3.0 programme [32]. Recruitment and data collection through questionnaires were conducted between 2014 and 2016. The start date of the study was 2014 because the BCSP in the Basque Country reached the whole target population (approximately 586,700 people) at the beginning of this year [31].

The characteristics of the sampling have been also described before [33]. Briefly, all the patients who were newly diagnosed with CRC (n = 601) were invited to participate in this study. Of those, 283 refused to participate in the study, and 10 were excluded due to missing information. Ultimately, 308 subjects (66.2% men) consented to participate in the survey and completed all the questionnaires. In addition, for each case, three age- and sex-matched control patients were randomly sought from the list of CRC-free subjects (n = 1836) who participated in the BCSP during the same period as the cases. The matched controls were patients with positive results (abnormal) for immunochemical fecal occult blood test (iFOBT) and negative colonoscopy results (normal). Finally, the matched case-to-control ratio was 1:1, and the final data set included 308 cases who were diagnosed with CRC and 308 age- and sex-matched controls. The descriptive socio-demographic and clinical data of the participants (cases vs controls) are shown in Supplementary Table S1.

The disease-related characteristics of the cases (pathological staging, location of cancer, tumour grade, and treatments) have been also described before [34]. Briefly, 72% were diagnosed with early-stage (I/II) CRC and 76% had a distal location of the cancer. This study was conducted according to the guidelines laid down in the Declaration of Helsinki, and all procedures involving patients were approved by the Clinical Research Ethics Committee of the Basque Country (reference numbers PI2011006 (approval date: 03/23/2012) and PI2014042 (approval date: 05/28/2014). Written informed consent was obtained from all the study participants. Consenting participants self-completed and returned one general questionnaire and a short food frequency questionnaire (FFQ). The questions referred to behaviours before participating in the BCSP. Assistance from the study staff was available to help the patients to understand the items on the questionnaires.

### Nutrient intake

The FFQ used to assess dietary intake was a modified version of the Rodríguez et al. questionnaire [35]. This adaptation was validated with multiple 24-h recalls in the Basque general population [36] and in CRC-diagnosed patients in a pilot of the present study [37]. It consisted of 67 items and requires the subjects to recall the number of times each food item was consumed either per week or per month. Moreover, the respondents could also record the consumption of other foods that were not included on the food list, as well as the use of dietetic products and nutritional supplements (generic and brand-name, dose, and frequency).

Once the completed FFQ was received, it was reviewed by a dietitian. Consumption frequencies were standardized to “per day” (/d) and multiplied by standard serving sizes (g or ml) [38]. For items that included several foods, each food’s contribution was estimated with weighting coefficients that were obtained from the usual consumption data [39]. All food items that were consumed were entered into DIAL 2.12 (2011 ALCE INGENIERIA) [40], a type of dietary assessment software, to estimate energy intake (kcal/d), vitamins related to OCM (vitamin B_2_, B_3_, B_6_, folate, and vitamin B_12_). The intakes of other methyl-donor compounds, in particular, Met, betaine, and total choline were estimated using the United States Department of Agriculture (USDA) Food Data central database [41] and from the publication of Zeisel et al. [42]. The data on nutrient intake presented in this study include daily intake from diet and dietetic products and supplements.

Because the nutrients, that are the object of this study, have all been considered to contribute to DNA methylation [43] and as other authors have done in previous studies [44], a composite “methyl-donor index” measure was derived by summing standardized intake values on the log scale [(value − mean)/SD] across the 8 individual nutrients. The nutritional characteristics of the diet of cases and controls have been described before [33]. Briefly, the diet was characterised by a low amount of carbohydrates (mean (SD), 36.5 (5.3)% of the total energy intake, in the total sample), but high amounts of fat (42.3 (4.8)% of the total energy intake, in the total sample), as well as by low folate intakes (48.7 (13.0)% of estimated average requirements, in the total sample). The diet followed by both cases and controls was a typical diet, participants in this study did not receive personalized dietary advice.

### Covariates

In addition to information on dietary intake, using a self-reported general questionnaire data on weight status (weight and height), environmental factors (demographic factors: age and sex; and lifestyle information: physical exercise (PE) and smoking habit) were recorded. These questions were taken from the Spanish Health Questionnaire [45]. Body mass index (BMI), estimated from self-reported height and weight, was classified according to the WHO criteria for those under 65 years of age [46] and according to the criteria proposed by Silva Rodríguez et al. for those 65 and older [47].

The FFQ used to assess dietary intake included specific questions about the frequency of intake of the following five major types of alcoholic beverages: beer, wine, cider, aperitif with alcohol, and liquor. These consumption frequencies were standardized to “per day” (/d) and multiplied by standard serving sizes (ml) [38]. The alcohol consumption data were expressed as grams of alcohol/d and were estimated with the software DIAL 2.12 (2011 ALCE INGENIERIA) [40]. From the data on daily alcohol consumption expressed in grams/d, standard drink units (SDU) were calculated, using the SDU defined for Spain (one SDU is the equivalent of 10 g of alcohol) [48].

The differences in general characteristics (age, BMI, PE, smoking habit, and alcohol consumption, among others) between cases and controls were previously described [34, 49]. Briefly, significant differences between cases and controls were found for smoking and weight status, with a higher percentage of cases with past or current smoking status and with overweight/obesity compared to controls (*p* < 0.01). However, no significant differences were found in alcohol consumption between cases and controls (*p* > 0.05).

Additionally, in both cases and controls, socio-economic level and health status (specifically health resource consumption) data were assessed with two indices that were obtained from the clinical databases developed by the Health Department of the Basque Government, namely the socioeconomic deprivation index (DI) and predictive risk modelling (PRM), respectively. The first one was estimated using the MEDEA project criteria [50], as has been described elsewhere [49], and was divided into quintiles (Q), with the first being the least disadvantaged and the fifth being the most disadvantaged. The PRM is an index that is based on Adjusted Clinical Groups (ACG) [51], Diagnostic Cost Groups/Hierarchical Condition Categories (DCG-HCC) [52], and Clinical Risk Groups (CRG) [53]. This index combines information about diagnoses, prescriptions, previous costs, and the use of specific procedures. It is capable of predicting the use of health resources [54], and it was stratified into four levels (L): the first included participants with a risk of high health resource consumption, and the fourth included those with low health resource consumption. The differences in these two indices (DI and PRM) between cases and controls were previously described [34]. Briefly, significant differences between the cases and the controls were found for DI and PRM, with a higher percentage of controls than cases in Q1-3 (the least disadvantaged) for DI, and a higher percentage of cases than controls in L1-2 (these levels included those with a risk of high health resource consumption) for PRM (*p* < 0.001).

### Statistical analysis

Statistical analyses for the present paper were performed using IBM SPSS Statistics for Windows, version 22.0 (IBM Corp., Armonk, NY, USA) and STATA 16.0 (StataCorp LP, Texas, USA). Continuous variables are shown as the means, standard deviation (SD), median, and 25 and 75 percentiles. Normality was checked using the Kolmogorov–Smirnov–Lilliefors test. Paired *t*-test or the Wilcoxon rank-sum test was used for two related means comparison. Conditional logistic regression was used to calculate odds ratios (ORs) and 95% confidence intervals (95%CI) for CRC risk according to tertiles of methyl-donor nutrient intakes for unadjusted (model I) and adjusted models (model II and III), for the total sample and, in addition, for stages I–II and distal localization (since they represent the most numerous subgroups according to the stage of diagnosis and location of the tumour, respectively). Models II and III were adjusted for age, sex, weight status (BMI), energy intake, PE level, smoking status, intensity of smoking, (in current and past smokers), time not smoking (in past smokers), alcohol intake (in SUD/d), socio-economic level (DI) and health status (PRM). In the model II, nutrient intakes were included separately, whereas in model III, all the nutrients related to OCM were included at the same time.

Intake of methyl-donor nutrients was categorized into tertiles (T) by the distribution in the control population, taking into account sex differences when they were significant (significant differences by sex were only observed for Met and alcohol intake). The lowest tertile was used as the reference group. Based on known risk factors for CRC [55–57], covariates in adjusted models included age, sex, weight status, energy intake, PE level, smoking status, the intensity of smoking (in current and past smokers), time not smoking (in past smokers), alcohol consumption, socioeconomic level, and health status. The reference categories were those that, according to the literature, have a lower CRC risk. In addition, to study the possible association between the intake of betaine and total choline, and the CRC risk, model II (adjusted model) of the regression analysis was also applied, including folate as covariate (these data are shown in text form in the Results section).

Quantitative covariates (cigarettes/d and years not smoking) were dichotomized by mean or median, according to the normality test. We used the cut-off of Romaguera et al. [58] to create two PE levels expressed in min/d of cycling/sports: sedentary-light (< 15 min/d) and moderate-vigorous (≥ 15 min/d). Age was dichotomized using the same age ranges that were used in the sample selection process (50–59 years old vs 60- 69 years old). Alcohol consumption variable (expressed as SUD/d) was categorized into tertiles by the distribution in the control population, considering sex differences since they were significant (men: T1 < 0.36, T2: 0.36–1.13; T3: > 1.13; women: T1 < 0.08, T2: 0.8–0.69, T3: > 0.69). Qualitative ones, such as DI and PRM were dichotomized considering the distribution of frequencies to obtain similar sample sizes for each category (DI, Q1-3 vs Q4-5; PRM, L3-4 vs L1-2). Energy intake was included as a quantitative variable in the adjusted models. We included participants with missing data for the covariates as a separate category. All tests were 2-sided, and *p*-values less than 0.05 were considered statistically significant.

## Results

Daily intake of OCM nutrients according to case–control status are shown in Supplementary Table S2. No significant differences were found between the two groups for most nutrients analysed, except for a higher consumption of Met, total choline, and betaine in the cases than the controls (*p* < 0.05). The separate analysis by sex of Met, total choline and betaine intake also showed significant differences between cases and controls (results not show). Regarding the nutrient intakes of cases according to tumour stage and their matched controls, nutrient intakes were not substantially different compared to their matched controls, except for Met, total choline, and betaine. The intake of these nutrients was lower in cases diagnosed in stages I–II (n = 222) in comparison with their matched controls (Met, 1700.5(SD 682.8) vs 1898.1(SD 758.9) mg/d, *p* = 0.010; total choline, 152.7(SD 105.2) vs 181.3(96.4) mg/d, *p* < 0.001; betaine, 108.6(SD 57.0) vs 150.1(SD 59.0) mg/d, *p* < 0.001). This last result was also observed when cases diagnosed in stages III-IV (n = 86) were compared with their matched controls (betaine, 108.6(SD 57.0) vs 150.1 (SD 59.0) mg/d, *p* < 0.001).

Major food sources for betaine and total choline, among control subjects are shown in Supplementary Table S3. Animal-based foods accounted for 69.1% of the total choline, and of these foods, the main sources of choline were eggs, sausage, and tuna, which together accounted for 43.9% of the total choline. Vegetables, cereals, and tubers were the main food sources of betaine, these products accounted for 81.6% of the total betaine. The ORs for CRC risk by the intake of nutrients related to OCM are presented in Table 1. The adjusted ORs for CRC risk decreased with higher intakes of betaine and total choline (*p* < 0.05). After further adjustment for folate, high intake of betaine and total choline remained associated with a reduced risk of CRC (Model II including folate as a covariate for total choline, OR_T3 vs T1_ = 0.45, 95% CI 0.26–0.80, *p* = 0.006; for betaine, OR_T3 vs T1_ = 0.27, 95% CI 0.16–0.47, *p* < 0.001).

**Table 1:** Association between the intake of nutrients related to one-carbon metabolism and colorectal cancer risk

Nutrients^a^	Case/control (n)	Model I^b^		Model II^c^		Model III^d^	
		OR (95% CI)	*p*^*e*^	OR (95% CI)	*p*^*e*^	OR (95% CI)	*p*^*e*^
Vitamin B_2_							
T1	118/108	1.00	-	1.00	-	1.00	-
T2	89/102	0.81(0.56–1.58)	0.272	0.61(0.35–1.06)	0.079	0.70(0.37–1.35)	0.292
T3	101/98	0.94(0.64–1.39)	0.774	0.72(0.38–1.33)	0.292	0.87(0.37–2.04)	0.752
Vitamin B_3_							
T1	103/101	1.00	-	1.00	**-**	1.00	-
T2	83/105	0.77(0.51–1.17)	0.220	0.67(0.37–1.24)	0.202	0.70(0.32–1.52)	0.366
T3	122/102	1.16(0.78–1.72)	0.463	1.02(0.54–1.93)	0.948	1.20(0.46–3.09)	0.709
Vitamin B_6_							
T1	94/89	1.00	-	1.00	-	1.00	-
T2	127/113	1.05(0.72–1.53)	0.807	0.80(0.46–1.38)	0.418	1.07(0.51–2.23)	0.862
T3	87/106	0.76(0.50–1.16)	0.208	0.52(0.28–0.98)	**0.048**	0.57(0.21–1.57)	0.280
Folate							
T1	112/102	1.00	-	1.00	-	1.00	-
T2	103/104	0.90(0.62–1.32)	0.588	0.73(0.44–1.21)	0218	0.77(0.41–1.43)	0.405
T3	93/102	0.84(0.57–1.22)	0.361	0.71(0.41–1.22)	0.212	0.95(0.45–2.03)	0.903
Vitamin B_12_							
T1	86/102	1.00	-	1.00	-	1.00	-
T2	108/103	1.24(0.84–1.83)	0.286	1.37(0.81–2.34)	0.245	1.43(073–2.78)	0.296
T3	114/103	1.32(0.89–1.96)	0.175	1.60/0.83–3.08)	0.161	1.50(0.63–3.60)	0.362
Met							
T1	111/102	1.00	-	1.00	-	1.00	-
T2	112/104	0.98(0.67–1.42)	0.910	0.92(0.56–1.49)	0.733	0.97(0.57–1.64)	0.07
T3	85/102	0.77(0.52–1.14)	0.194	0.54(0.30–0.95)	**0.032**	0.61(0.33–1.12)	0.108
Choline							
T1	147/103	1.00	-	1.00	**-**	1.00	-
T2	99/102	0.70(0.48–1.02)	0.063	0.58(0.34–0.98)	**0.044**	0.62(0.36–1.08)	0.090
T3	62/103	0.42(0.28–0.64)	** < 0.001**	0.46(0.26–0.80)	**0.006**	0.49(0.28–0.89)	**0.019**
Betaine							
T1	194/102	1.00	-	1.00	**-**	1.00	**-**
T2	64/104	0.34(0.22–0.51)	** < 0.001**	0.44(0.26–0.76)	**0.003**	0.46(0.027–0.80)	**0.004**
T3	50/102	0.28(0.18–0.43)	** < 0.001**	0.29(0.17–0.49)	** < 0.001**	0.030(0.18–0.49)	** < 0.001**
*Methyl donor index*^*f,g*^							
T1	155/102	1.00	-	1.00	**-**		
T2	112/103	0.76(0.51–1.12)	0.169	0.60(0.36–1.01)	0.055		
T3	41/103	0.86(0.59–1.25)	0.423	0.28(0.15–0.52)	** < 0.001**		

^a^Nutrients related to one-carbon metabolism were categorized into tertiles according to the distribution in controls; Tertiles of vitamin B_2_ (mg/d): T1 < 1.4, T2 1.4–1.7, T3 > 1.7; vitamin B_3_ (mg/d): T1 < 26.5, T2 26.5–32.4, T3 > 32.4; vitamin B_6_ (mg/d): T1 < 1.6, T2 1.6–2.0, T3 > 2.0; folate (µg/d): T1 < 238.0, T2 238.0–298.0, T3 > 298.0; vitamin B_12_ (µg/d): T1 < 4.0, T2 4.0–5.3, T3 > 5.3; methyl donor index: T1 < −4.46, T2 −4.46 to −3.47, T3 > −3.47; Met (mg/d) for men: T1 < 1377.0, T2 1377.0–1985.0, T3 > 1985.0; Met (mg/d) for women: T1 < 1665.0, T2 1665.0–2531.0, T3 > 2531.0; total choline (mg/d): T1 < 117.0, T2 117.0–187.0, T3 > 187.0; betaine (mg/d): T1 < 119.0, T2 119.0–170.0, T3 > 170.0

^b^Model I, analyses were performed using crude conditional logistic regression, without considering confounding factors

^c^Model II, analyses were performed using conditional logistic regression analysis adjusted for age (50–59 years old, 60–69 years old), sex, body mass index (underweight/normal weight, overweight/obesity), energy intake (kcal/d), physical exercise level (< 15 min/d of cycling/sports, ≥ 15 min/d), smoking status and intensity of smoking (never; past: quit smoking ≥ 11 years ago, quit < 11 years ago; smoker: ≤ 15 cigarettes/d, > 15 cigarettes/d), alcohol intake (standard unit drinks/d, tertiles in controls: men: T1 < 0.36, T2 0.36–1.13, T3 > 1.13; women: T1 < 0.08, T2 0.8–0.69, T3 > 0.69), Deprivation Index (quintile 1–3, quintile 4–5) and Predictive Risk Modelling (level 1–2, level 3–4), including nutrient intakes separately; participants with missing data for the confounding variables were included as a separate category for these variables

^d^Model III, model II including all the nutrients related to one-carbon metabolism

^e^A value of *p* < 0.05 was considered significant. Significant results are highlighted in bold

^f^Model III is not applicable because this index includes all nutrients in its construction

^g^This index was derived by summing standardized intake values on the log scale [(value − mean)/SD] across the 8 individual nutrients

*CI* Confidence interval, *Met* Methionine, *OR* Odds ratio, *T* Tertile

No significant differences were observed in the methyl-donor index between cases and controls (*p* > 0.05). In this same line, the results of the conditional logistic regression analysis were not significant in model I; however, the adjusted ORs for CRC risk decreased with higher scores in the methyl-donor index (Model II, OR_T3 vs T1_ = 0.28, 95% CI 0.15–0.52, *p* < 0.001). Separate analyses by sex showed that adjusted ORs for CRC risk did not decrease with higher intakes of choline in model II for men, and in model III neither for men nor for women (*p* > 0.05) (Supplementary Table S4 and S5). However, the results for the methyl-donor index presented that un- and adjusted ORs for CRC risk decrease with higher scores, both in men (*p* < 0.001) and women (*p* < 0.05).

The ORs for CRC risk decreased with higher intakes of betaine for cases diagnosed in stages I-II (Model I, OR_T3 vs T1_ = 0.23, 95%CI: 0.13–0.43, *p* < 0.001; Model II, OR_T3 vs T1_ = 0.29, 95% CI 0.17–0.49, *p* < 0.001; Model III, OR_T3 vs T1_ = 0.30, 95% CI 0.18–0.49, *p* < 0.001). Due to the small sample size, adjusted regression models could not be calculated for cases diagnosed in stages III-IV; in any case, the crude conditional logistic regressions for these stages did not show significant associations between the intake of OCM nutrients and CRC risk, except vitamin B_6_ (Model I, OR_T3 vs T1_ = 0.18, 95% CI 0.04–0.91, *p* < 0.05).

Significant differences in intakes of betaine and total choline were also observed between cases and controls when the cases were divided according to the tumour location. Said intakes were lower both in cases with proximal localization (n = 74) than in those with distal localization (n = 234) in comparison with their matched controls (total choline: cases with proximal localization, 136.4(SD 86.4) vs matched controls, 171.4(SD 87.6) mg/d, *p* = 0.012; cases with distal localization, 134.1(SD 85.2) vs matched controls, 160.6(SD 84.2) mg/d, *p* < 0.001; betaine: cases with proximal localization, 109.0(SD 53.5) vs matched controls, 115.1(SD 55.7) mg/d, *p* < 0.001; cases with distal localization, 141.0 (SD 64.7) vs matched controls 154.1(SD 62.1) mg/d, *p* < 0.001). These results were confirmed in the regression analyses carried out in cases with distal localization. Due to the small sample size, adjusted regression models could not be calculated for cases with proximal localization.

The ORs for CRC risk decreased with higher intakes of choline in cases with distal localization in models I and II for T2 (Model I, OR_T2 vs T1_ = 0.47, 95% CI 0.27–0.84, *p* = 0.011; Model II, OR_T2 vs T1_ = 0.44, 95% CI: 0.30–0.75, *p* = 0.001) and in all models for the T3 (Model I, OR_T3 vs T1_ = 0.25, 95% CI: 0.12–0.44, *p* < 0.001; Model II, OR_T3 vs T1_ = 0.71, 95% CI: 0.49–0.98, *p* = 0.043; Model III, OR_T3 vs T1_ = 0.40, 95% CI 0.25–0.63, *p* < 0.001). In addition, the ORs for CRC risk decreased with higher intakes of betaine in cases with distal localization in all models for the T2 (Model I, OR_T2 vs T1_ = 0.31, 95% CI 0.19–0.51, *p* < 0.001; Model II, OR_T2 vs T1_ = 0.44, 95% CI 0.26–0.76, *p* = 0.003; Model III, OR_T2 vs T1_ = 0.46, 95% CI 0.27–0.80, *p* = 0.004) and for the T3 (Model I, OR_T3 vs T1_ = 0.29, 95% CI 0.17–0.42, *p* < 0.001; Model II, OR_T3 vs T1_ = 0.65, 95% CI 0.45–0.98, *p* = 0.044; Model III, OR_T3 vs T1_ = 0.45, 95% CI 0.20–0.62, *p* < 0.001).

## Discussion

In this case–control study, a significant inverse association was found between dietary intake of betaine and total choline, and the risk of CRC. Sub-group analysis for stages I–II and distal localization of tumour also showed lower intakes of these nutrients, total choline, and betaine, in cases in comparison with their matched controls. However, no association was found between the intake of other nutrients related to OCM (such as folate, vitamins of group B, or Met) and CRC risk, and consequently, neither between the methyl-donor index and CRC risk (at least for the total sample).

To date, few epidemiologic studies have examined the association between total choline and betaine and cancer risk, and those who have studied this possible relationship have obtained inconsistent results. Several researchers found an inverse association between total choline and betaine intake and breast cancer risk [59, 60]. However, other studies found no evidence that higher intakes of these nutrients reduced the risk of breast cancer [61, 62]. Some studies have reported that higher intakes of choline and betaine were associated with a reduced risk of lung cancer [63] and nasopharyngeal carcinoma [64], whereas no association was found for epithelial ovarian cancer [65].

Inconsistent results were also observed on the relationship between total choline and betaine intake and CRC risk. The Health Professionals Follow-up Study conducted in the United States [26] and an investigation carried out in a Chinese population [66] have examined this possible association, and in both studies, no association was found. This result has attributed, in part, to the fact that the intake of these nutrients would not be critical in folate-nourished populations (because folate and choline metabolic pathways are highly interrelated) [67]. However, the Nurses´ Health Study [68] showed that increasing choline intake was associated with an elevated risk of colorectal adenoma in women. In contrast, our results about choline were consistent with the findings of Lu et al. [66], showing that increased intakes of choline reduce the CRC risk (in the total sample and women separately), even among subjects with an average total folate intake below population recommendations and below the intakes recorded in other studies, such as the Health Professionals Follow-up Study mentioned above [26] (with a total folate intake from diet and supplements of 479–858 µg/day in the total sample).

In the present study, the average folate intake was 273.3 µg/d among controls and therefore the intake level of folate was not very high. A previous study has shown that choline can be utilized as a methyl-donor when folate intake is low [69]. Therefore, relatively lower dietary folate intake in the present research could contribute to the detection of the protective effect of choline intake on CRC risk.

Similar associations are expected between CRC risk and choline intake as with betaine intake, particularly as betaine is derived from choline and since betaine levels increase in response to higher choline intake [70]. These associations agree with the findings of a case–control study nested within the EPIC cohort, where subgroup analyses of women found plasma choline was inversely associated with CRC risk [71], together with results of other case–control studies where plasma betaine was inversely associated with CRC risk [72, 73]. That said, the few studies published on the association between betaine intake and CRC risk are inconclusive. Neither the Health Professionals Follow-up Study [26] nor the study carried out by Lu et al. [66], found significant associations between CRC risk and betaine intake. These discrepancies could be due to differences in the study design type, characteristics of participants, their dietary intakes, and the status of other nutrients involved in OCM. Both were prospective cohort studies but the Health Professionals Follow-up Study comprised US males aged 40–75 years with folate intakes between 479 and 858 µg/day [26], whereas, Lu et al. represents Chinese males and females aged 30–75 years, with an average folate intake of 240.3 µg/d [66].

The different intake levels and food sources of choline and betaine in the aforementioned studies might also explain the discrepancies. The mean choline and betaine intake in the control group of the present study were 163.2 mg/d and 151 mg/d, respectively, which were comparable with the results of a study carried out in a Chinese population [66]. In the Nurses´ Health Study [68], betaine levels of 189 mg/d were relatively high compared to our results, but mean choline intake was practically doubled, measured at 331 mg/d.

Regarding the other nutrients related to OCM (such as vitamins of the B group), our findings indicated a non-significant decrease in CRC risk with the high level of intake. These data agree with those of one meta-analysis of prospective cohort studies on vitamin B_6_ and CRC risk [19], but they contrast with a dose–response meta-analysis about vitamin B_2_ and CRC risk [74]. Next to different sample sizes among studies, this inconsistency may also have been caused by possible selection bias that is likely to occur in case–control studies but not in cohort studies, potential differences in the level of over- or underestimation of dietary intake using FFQ, and different adjustments for confounding factors between studies [75]. In addition, the inconsistencies may be due to differences between study populations and inherent variations in levels of nutrient intake.

In any case, the importance of nutrients related to OCM in cancer prevention, and its role in colorectal carcinogenesis is still a subject of debate. So, more studies are needed to understand and identify risk and protective factors and to better characterise specific effects, according to sex, site, and stage tumour, as well as to variation in DNA methylation, for a better prevention approach.

The main strength of this study compared to others [76, 77] is that colonoscopy was used as a diagnosis criterion to identify both cases and controls, to avoid false positives and negatives. To our knowledge, to date only one other study of the association between diet and CRC risk has been published, in which it was confirmed that controls were free of the disease through colonoscopy [78]. Another strength is the fact that information is provided based on a standardised protocol including not only dietary factors but also other possible determinants of CRC such as health determinants and weight status among others. However, some limitations should be mentioned. First, recall bias is also of concern in case–control studies. Second, self-reported data could be subject to measurement errors and the problem of food omissions due to memory failure and under-reporting of unhealthy habits among disease subjects. However, previous validation studies indicate that self-reported dietary information is reported with sufficient accuracy for use in epidemiology analysis [79]. Third, although the FFQ used to collect information on dietary intake in the present study has been validated among people who lived in the same region, this validation did not include specific nutrients such as betaine or total choline, but it included the main food sources of these methyl-donors. So, that the possible measurement errors are most likely non-differential and thus do not explain the inverse associations observed in our study. Fourth, even though data on lifestyles (including diet) were recorded retrospectively—that is, the questions referred to behaviours before participating in the BCSP—it should be also noted that dietary changes are usually modest after participating in the BCSP due to a lack of information and personalized advice [80, 81]. In addition, adults generally maintain a relatively stable eating habit for a long time [82]. Therefore, the results of this study are unlikely to be greatly affected by potential changes in eating. Fifth, although we have adjusted for several confounding factors, some residual confounding may result from the misclassification of those variables and confounding by unmeasured variables. Finally, to avoid selection bias of controls, we obtained controls from the same BCSP and in the same period as cases; thus, it was confirmed that they did not suffer from CRC by colonoscopy.

In conclusion, although no significant association between nutrients related to OCM, such as folate and vitamins of group B, and CRC risk was found, our data suggest that betaine and total choline intake influence CRC risk in the studied population. Further studies are necessary to confirm this inverse association and understand in depth its role in colorectal carcinogenesis.

### Supplementary Information

Below is the link to the electronic supplementary material.

Supplementary file1 (PDF 358 KB)

## Data Availability

Data described in the manuscript will be made available upon request and approval from the corresponding author.
