# Supplementary material for: Association between nutrient intake related to the one-carbon metabolism and colorectal cancer risk: a case–control study in the Basque Country
Source: Eur J Nutr. 2023 Aug 6;62(8):3181–91. doi: 10.1007/s00394-023-03229-2 (PMC10611602; doi:10.1007/s00394-023-03229-2)
Supplement: Supplementary file 1 — Supplementary file1 (PDF 358 KB) [file 394_2023_3229_MOESM1_ESM.pdf]

Association between nutrient intake related to the one-carbon metabolism and colorectal cancer risk: a case-control study in the Basque Country

Silvia Martín-Fernández-de Labastida, Iker Alegria-Lertxundi, Marian M. de Pancorbo, Marta Arroyo-Izaga*

***Correspondence: Marta Arroyo-Izaga, BIOMICs Research Group, Microfluidics & BIOMICs Cluster, Department of Pharmacy and Food Sciences, University of the Basque Country UPV/EHU, 01006 Vitoria-Gasteiz, Araba/Álava, Spain; Bioaraba, BA04.03, Vitoria-Gasteiz, Araba/Álava, Spain. marta.arroyo@ehu.eus

Supplementary Table S1. Descriptive socio-demographic and clinical data of the participants (cases vs controls)

Characteristics	Cases (n=308)		Controls (n=308)		*p*
Sex, % of men	66.2		66.2		
Age, y, mean SD	61.5	5.2	61.1	5.5	0.093
Smoking status, %					
Never	27.9		38.6		
Past/current	72.1		61.4		0.004
BMI, %					
Underweight	6.5		7.8		
Normal weight	26.0		34.1		
Overweight/obesity	67.5		58.1		0.033
PRM, %[1]					
L_{1-2}	15.6		12.3		
L_{3-4}	83.4		79.2		<0.001
DI, %[1]					
Q_{1-3}	47.1		65.6		
Q_{4-5}	18.8		29.5		<0.001

[1]Valid percentages

BMI body mass index, *DI* deprivation index (this index was successfully assigned to 80.2% of the study sample), *L* level, *PRM* predictive risk modelling (this index was successfully assigned to 95.1% of the study sample), *Q* quintile, *SD* standard deviation

Supplementary Table S2. Daily intake of one-carbon metabolism nutrients in cases and control studied

Daily nutrient intake	Cases (n=308)					Controls (n=308)					
	Mean	SD	Median	P25	P75	Mean	SD	Median	P25	P75	p^a
Vitamins related to OCM											
Vitamin B_2, mg/d	1.5	0.5	1.4	1.2	1.7	1.6	0.5	1.5	1.2	1.9	0.100
Vitamin B_3, mg/d	30.1	7.1	30.2	24.3	35.0	30.3	7.7	29.7	25.2	34.0	0.893
Vitamin B_6, mg/d	2.7	14.3	1.8	1.5	2.1	1.9	0.6	1.8	1.5	2.2	0.378
Folate, µg/d	267.2	80.1	261.0	209.0	307.0	273.3	76.5	261.5	217.3	325.0	0.406
Vitamin B_{12}, µg/d	6.6	28.5[b]	4.8	3.8	6.0	4.9	1.7	4.5	3.7	5.8	0.094
Other dietary methyl donors											
Methionine, mg/d	1753.7	644.6	1717.5	1218.5	2146.3	1894.2	747.6	1810.5	1255.8	2473.00	**0.012**
Choline, mg/d	134.7	85.4	120.5	80.8	170.0	163.2	85.0	147.0	99.3	212.8	**<0.001**
Betaine, mg/d	113.6	55.2	101.5	71.4	144.8	151.0	62.9	141.5	102.0	187.8	**<0.001**
Methyl donor index[c]	-4.1	1.3	-4.1	-4.9	-3.2	-4.0	1.3	-4.0	-4.8	-3.1	0.653

[a]Differences between cases and controls. A value of $p < 0.05$ was considered significant. Significant results are highlighted in bold
[b]This high SD is due to the use of a nutritional supplement by one of the cases
[c]This variable follows a normal distribution (the rest of the variables follow a non-normal distribution). This index was derived by summing standardized intake values on the log scale [(value − mean)/SD] across the 8 individual nutrients
Met Methionine, *OCM* one-carbon metabolism, *P25* 25 percentile, *P75* 75 percentile, *SD* standard deviation

Supplementary Table S3. Top five food sources of total choline and betaine among control subjects

Food sources	Proportion
Total choline	
Eggs	24.9
Sausage	7.9
Lentils	7.9
Beef	5.8
Tuna	5.3
Betaine	
Pasta	48.1
Green beans	17.5
Potatoes	11.1
Beef	8.5
Lettuce	4.9

Supplementary Table S4. Association between the intake of nutrients related to one-carbon metabolism and colorectal cancer risk in men

Nutrients[a]	Case/control(n)	Model I[b]		Model II[c]		Model III[d]	
		OR (95% CI)	p^e	OR (95% CI)	p^e	OR (95% CI)	p^e
Vitamin B_2							
T1	89/82	1.00	-	1.00	-	1.00	-
T2	55/58	0.88(055–1.40)	0.586	0.61(0.33–1.14)	0.122	0.50(0.21–1.16)	0.108
T3	60/64	0.86(0.54–1.38)	0.535	0.47(0.22–0.99)	0.046	0.59(0.20–1.79)	0.355
Vitamin B_3							
T1	72/73	1.00	-	1.00	-	1.00	-
T2	56/59	0.97(0.58–1.61)	0.897	1.09(0.54–2.21)	0.813	1.01(0.37–2.77)	0.986
T3	76/72	1.07(0.66–1.73)	0.779	1.00(0.55–1.83)	0.995	1.05(0.31–3.56)	0.938
Vitamin B_6							
T1	69/70	1.00	-	1.00	-	1.00	-
T2	81/63	1.28(0.80–2.05)	0.308	0.96(0.51–1.81)	0.891	0.81(0.30–2.18)	0.680
T3	54/71	0.75(0.45–1.25)	0.266	0.61(0.30–1.25)	0.176	0.71(0.18–2.80)	0.625
Folate							
T1	84/73	1.00	-	1.00	-	1.00	-
T2	66/62	0.92(0.58–1.45)	0.704	0.88(0.49–1.56)	0.655	0.80(0.37–1.76)	0.585
T3	54/69	0.69(0.43–1.11)	0.123	0.67(0.36–1.26)	0.220	1.05(0.31–3.56)	0.955
Vitamin B_{12}							
T1	54/68	1.00	-	1.00	-	1.00	-
T2	73/68	1.33(0.82–2.14)	0.248	1.28(0.70–2.33)	0.422	1.58(0.66–3.80)	0.302
T3	77/68	1.41(0.87–2.30)	0.162	1.85(0.85–4.01)	0.119	2.62(0.88–7.74)	0.082
Met							
T1	70/68	1.00	-	1.00	-	1.00	-
T2	66/69	0.93(0.58–1.50)	0.768	0.87(0.48–1.58)	0.646	0.86(0.42–1.78)	0.692
T3	68/67	0.98(0.60–1.61)	0.942	0.67(0.34–1.32)	0.251	0.72(0.32–1.64)	0.434

Choline							
T1	92/71	1.00	-	1.00	-	1.00	-
T2	68/73	0.72(0.46–1.13)	0.155	0.77(0.43–1.38)	0.379	0.74(0.35–1.55)	0.421
T3	44/60	0.55(0.33–0.92)	**0.024**	0.64(0.34–1.22)	0.174	0.75(0.34–1.64)	0.469
Betaine							
T1	122/65	1.00	-	1.00	-	1.00	-
T2	46/67	0.39(0.23–0.64)	**<0.001**	0.62(0.34–1.15)	0.128	0.58(0.28–1.21)	0.149
T3	36/72	0.29(0.18–0.49)	**<0.001**	0.30(0.16–0.56)	**<0.001**	0.27(0.13–0.58)	**<0.001**
Methyl donor index[f,g]							
T1	98/61	1.00	-	1.00	-	-	-
T2	75/71	0.63(0.40–1.02)	0.058	0.42(0.22–0.78)	**0.006**	-	-
T3	31/72	0.28(0.16–0.48)	**<0.001**	0.19(0.09–0.41)	**<0.001**	-	-

[a]Nutrients related to one-carbon metabolism were categorized into tertiles according to the distribution in controls; Tertiles of vitamin B_2 (mg/d): T1 < 1.4, T2 1.4–1.7, T3 > 1.7; vitamin B_3 (mg/d): T1 < 26.5, T2 26.5–32.4, T3 > 32.4; vitamin B_6 (mg/d): T1< 1.6, T2 1.6–2.0, T3 > 2.0; folate (µg/d): T1 < 238.0, T2 238.0–298.0, T3 > 298.0; vitamin B_{12} (µg/d): T1 < 4.0, T2 4.0–5.3, T3 > 5.3; methyl donor index: T1 < -4.46; T2 -4.46–-3.47, T3 > -3.47; Met (mg/d) for men: T1 < 1377.0, T2 1377.0–1985.0, T3 > 1985.0; Met (mg/d) for women: T1 < 1665.0, T2 1665.0–2531.0, T3 > 2531.0; total choline (mg/d): T1 < 117.0, T2 117.0–187.0, T3 > 187.0; betaine (mg/d): T1 < 119.0, T2 119.0–170.0, T3 > 170.0

[b]Model I, analyses were performed using crude conditional logistic regression, without considering confounding factors

[c]Model II, analyses were performed using conditional logistic regression analysis adjusted for age (50–59 years old, 60–69 years old), sex, body mass index (underweight/normal weight, overweight/obesity), energy intake (kcal/d), physical exercise level (< 15 min/d of cycling/sports, ≥ 15 min/d), smoking status and intensity of smoking (never; past: quit smoking ≥ 11 years ago, quit < 11 years ago; smoker: ≤ 15 cigarettes/d, > 15 cigarettes/d), alcohol intake (standard unit drinks/d, tertiles in controls: men: T1 < 0.36, T2 0.36–1.13, T3 > 1.13; women: T1 < 0.08, T2 0.8–0.69, T3 > 0.69), Deprivation Index (quintile 1–3, quintile 4–5) and Predictive Risk Modelling (level 1–2, level 3–4), including nutrient intakes separately; participants with missing data for the confounding variables were included as a separate category for these variables

[d]Model III, model II including all the nutrients related to one-carbon metabolism

[e]A value of $p < 0.05$ was considered significant. Significant results are highlighted in bold

[f]Model III is not applicable because this index includes all nutrients in its construction

[g]This index was derived by summing standardized intake values on the log scale [(value − mean)/SD] across the 8 individual nutrients

CI Confidence interval, *Met* Methionine, *OR* Odds ratio, *T* Tertile

Supplementary Table S5. Association between the intake of nutrients related to one-carbon metabolism and colorectal cancer risk in women

Nutrients[a]	Case/control(n)	Model I[b]		Model II[c]		Model III[d]	
		OR (95% CI)	p^e	OR (95% CI)	p^e	OR (95% CI)	p^e
Vitamin B_2							
T1	29/26	1.00	-	1.00	-	1.00	-
T2	34/44	0.73(0.38–1.42)	0.356	0.88(0.37–2.05)	0.760	3.63(0.06–221.1)	0.087
T3	41/34	1.10(0.55–2.21)	0.794	1.38(0.50–3.81)	0.533	37.2(0.04–31674.4)	0.085
Vitamin B_3							
T1	31/28	1.00	-	1.00	-	1.00	-
T2	27/46	0.49(0.23–1.05)	0.065	0.18(0.05–0.66)	0.010	0.02(0.00–0.62)	**0.027**
T3	46/30	1.36(0.66–2.80)	0.402	0.78(0.22–2.74)	0.693	1.77(0.07–41.72)	0.724
Vitamin B_6							
T1	25/19	1.00	-	1.00	-	1.00	-
T2	46/50	0.74(0.38–1.43)	0.367	0.55(0.22–1.33)	0.182	0.28(0.00–20.53)	0.563
T3	33/35	0.74(0.36–1.55)	0.430	0.48(0.18–1.33)	0.158	0.82(0.01–55.58)	0.928
Folate							
T1	28/29	1.00	-	1.00	-	1.00	-
T2	37/42	0.90(0.45–1.80)	0.769	0.45(0.17–1.15)	0.094	0.07(0.00–2.60)	0.150
T3	39/33	1.18(0.61–2.28)	0.622	0.67(0.27–1.64)	0.381	0.01(0.00–1.13)	0.056
Vitamin B_{12}							
T1	32/34	1.00	-	1.00	-	1.00	-
T2	35/35	1.07(0.54–2.13)	0.849	1.33(0.53–3.37)	0.542	0.04(0.00–1.39)	0.075
T3	37/35	1.13(0.57–2.25)	0.725	0.89(0.29–2.77)	0.844	0.01(0.00–1.94)	0.087
Met							
T1	41/34	1.00	-	1.00	-	1.00	-
T2	46/35	1.13(0.61–2.09)	0.708	1.14(0.54–2.42)	0.726	0.99(0.15–6.37)	0.992
T3	17/35	0.48(0.24–0.94)	0.034	0.31(0.11–0.86)	0.024	0.01(0.00–2.37)	0.098

Choline							
T1	55/32	1.00	-	1.00	-	1.00	-
T2	31/29	0.70(0.35–1.43)	0.330	0.35(0.12–0.98)	**0.046**	0.13(0.01–2.47)	0.176
T3	18/43	0.27(0.13–0.56)	**<0.001**	0.21(0.07–0.60)	**0.004**	1.57(0.09–26.34)	0.755
Betaine							
T1	72/37	1.00	-	1.00	-	1.00	-
T2	18/37	0.26(0.12–0.54)	**<0.001**	0.28(0.11–0.74)	0.011	0.01(0.00–1.59)	0.077
T3	14/30	0.26(0.12–0.56)	**<0.001**	0.18(0.06–050)	**0.001**	0.00(0.00–0.78)	**0.041**
Methyl donor index[f,g]							
T1	57/41	1.00	-	1.00	-	-	-
T2	37/32	0.92(0.46–1.86)	0.824	0.87(0.35–2.14)	0.762	-	-
T3	10/31	0.27(0.12–0.61)	**0.002**	0.27(0.09–0.77)	**0.015**	-	-

[a]Nutrients related to one-carbon metabolism were categorized into tertiles according to the distribution in controls; Tertiles of vitamin B_2 (mg/d): T1 < 1.4, T2 1.4–1.7, T3 > 1.7; vitamin B_3 (mg/d): T1 < 26.5, T2 26.5–32.4, T3 > 32.4; vitamin B_6 (mg/d): T1< 1.6, T2 1.6–2.0, T3 > 2.0; folate (µg/d): T1 < 238.0, T2 238.0–298.0, T3 > 298.0; vitamin B_{12} (µg/d): T1 < 4.0, T2 4.0–5.3, T3 > 5.3; methyl donor index: T1 < -4.46; T2 -4.46–-3.47, T3 > -3.47; Met (mg/d) for men: T1 < 1377.0, T2 1377.0–1985.0, T3 > 1985.0; Met (mg/d) for women: T1 < 1665.0, T2 1665.0–2531.0, T3 > 2531.0; total choline (mg/d): T1 < 117.0, T2 117.0–187.0, T3 > 187.0; betaine (mg/d): T1 < 119.0, T2 119.0–170.0, T3 > 170.0

[b]Model I, analyses were performed using crude conditional logistic regression, without considering confounding factors

[c]Model II, analyses were performed using conditional logistic regression analysis adjusted for age (50–59 years old, 60–69 years old), sex, body mass index (underweight/normal weight, overweight/obesity), energy intake (kcal/d), physical exercise level (< 15 min/d of cycling/sports, ≥ 15 min/d), smoking status and intensity of smoking (never; past: quit smoking ≥ 11 years ago, quit < 11 years ago; smoker: ≤ 15 cigarettes/d, > 15 cigarettes/d), alcohol intake (standard unit drinks/d, tertiles in controls: men: T1 < 0.36, T2 0.36–1.13, T3 > 1.13; women: T1 < 0.08, T2 0.8–0.69, T3 > 0.69), Deprivation Index (quintile 1–3, quintile 4–5) and Predictive Risk Modelling (level 1–2, level 3–4), including nutrient intakes separately; participants with missing data for the confounding variables were included as a separate category for these variables

[d]Model III, model II including all the nutrients related to one-carbon metabolism

[e]A value of $p < 0.05$ was considered significant. Significant results are highlighted in bold

[f]Model III is not applicable because this index includes all nutrients in its construction

[g]This index was derived by summing standardized intake values on the log scale [(value − mean)/SD] across the 8 individual nutrients

CI Confidence interval, *Met* Methionine, *OR* Odds ratio, *T* Tertile
